# Evaluation of the anti-biofilm effect of poloxamer-based thermoreversible gel of silver nanoparticles as a potential medication for root canal therapy

**DOI:** 10.1038/s41598-021-92081-7

**Published:** 2021-06-15

**Authors:** Ting Liu, Aerdake Aman, Muniremu Ainiwaer, Liang Ding, Fei Zhang, Qingang Hu, Yuxian Song, Yanhong Ni, Xuna Tang

**Affiliations:** 1 Nanjing Stomatological Hospital, Medical School of Nanjing University, Nanjing, People’s Republic of China; 2 Central Laboratory of Stomatology, Nanjing Stomatological Hospital, Medical School of Nanjing University, Nanjing, People’s Republic of China; 3 Present Address: Department of Endodontology, Nanjing Stomatological Hospital, No. 30 Zhongyang Road, Nanjing, People’s Republic of China

**Keywords:** Biochemistry, Drug discovery, Microbiology, Medical research

## Abstract

The purpose of this study was to design silver nanoparticles (AgNPs) poloxamer thermoreversible gel (AgNPs-PL) and investigate whether this gel could provide sustained antibacterial activity against *Enterococcus faecalis* (*E. faecalis*) in the root canal. The gels fabricated were characterized in terms of gelatin temperature, particle size, in-vitro Ag^+^ release, and elemental content. Cytotoxicity of AgNPs-PL on primary human periodontal ligament fibroblasts (HPDLFs) was examined by CCK-8 assay. Characterization of AgNPs-PL gel revealed that it contained particles existing as large clumps/fused aggregates of different shapes, with a mean diameter of 21.624 ± 14.689 nm, exhibited sustained release of Ag^+^ for 9 days, and non-toxic to HPDLFs at a low dose (4–32 μg/mL) through 24, 48, and 72 h exposures. The antibacterial effect of 16 and 32 μg/mL concentrations of AgNPs-PL was compared with blank poloxamer gel (PL) and calcium hydroxide (CH) using three methods: (I) agar counting plate, (II) scanning electron microscope (SEM) observations, and (III) confocal laser scanning microscope (CLSM) analysis. AgNPs-PL at the two doses above was more effective than PL and CH in removing *E. faecalis* biofilm at 1, 3, 9 days. Thus, AgNPs-PL exhibits strong activity against *E. faecalis* and is easy to produce, with a continuous release profile of Ag^+^. AgNPs-PL gel may be a candidate for a new root canal disinfection.

## Introduction

Bacterial biofilms comprise bacterial colonies, polysaccharides, and proteins, and often attach to organic surfaces. Biofilms provide a protective matrix that shields bacteria from antimicrobial agents and host defenses^1^. The ideal goal of root canal therapy is to completely eradicate biofilms and their by-products, thereby preventing reinfection^2^. However, root canal therapy may fail as a result of persistent and chronic endodontic infection or reinfection, which may be due to high microbial diversity, drug resistance by bacterial biofilms, and other factors such as the complex anatomy of the root canal^2,3^. *Enterococcus faecalis* (*E. faecalis*) is the most common bacterial infection leading to failed root canal therapy. *E. faecalis* is resistant to antimicrobial agents, starvation, high salt concentration, high pH value, and can penetrate dentinal tubules^4,5^. Owing to these characteristics, *E. faecalis* biofilm models are frequently used to evaluate the antimicrobial properties of disinfectants^6^.

In endodontic treatment, calcium hydroxide (CH) is most commonly used as intracanal medicament due to its antibacterial effects upon direct contact with microorganisms^7,8^. The antimicrobial activity of CH is associated with the high pH it generates by releasing hydroxyl ions^9^. However, previous studies indicate that CH is ineffective against *E. faecalis* in root canals as this microbe can withstand a pH as high 11.5^10^. Nanoparticles with diameters of less than 100 nm have been found to exert antibacterial activity against many species of bacteria^11^. When used as nanoparticles, some inorganic metals, like silver and gold, exhibit strong antibacterial properties^12–14^. For instance, silver nanoparticles (AgNPs) can kill numerous bacterial species and is therefore widely used in various agents for the disinfection of medical devices^15–17^. AgNPs have been reported to destabilize bacterial membranes, increasing their permeability, and thus resulting in bacterial membrane disintegration^18,19^. We had previously contemplated whether AgNPs could be used alone as endodontic antimicrobial agents for root canal therapy. However, a major challenge with this approach is that the AgNPs we routinely formulated was in liquid form and could therefore not be contained in the root canal for application. We, therefore, sought to identify a material that is capable of scaffolding AgNPs and slowly releasing silver ions over an extended period, thereby enhancing its antibacterial effects.

Poloxamers (PL), which consist of polyethylene oxide (PEO) and polypropylene oxide (PPO) units, possess high surfactant properties, relatively fast dissolution rate, and non-toxic^20^. Such polymers are therefore frequently used as drug delivery systems^21^. Poloxamers 407 (P407, or Pluronics F127) and Poloxamers188 (P188) are polymers commonly used to make thermoreversible gels^22,23^. While P407 improves bioavailability, P188 regulates thermal transition temperature^24–26^. Because of its ability to self-assemble into micelles, PL exhibits thermoreversible properties, which allows it to occur as a gel at temperatures close to body temperature while occurring in liquid form at room temperature^22^. In oral applications, thermoreversible poloxamer hydrogels have been used as carriers for sustained-release of various agents, thereby providing effective treatment for periodontal disease^27,28^. We reasoned that owing to its properties, poloxamer thermoreversible gel is well suited for application as a carrier of AgNPs for root canal therapy. We developed AgNPs poloxamer thermoreversible gel (AgNPs-PL), which improved the long-term antimicrobial effect of AgNPs. We analyzed the physicochemical properties of AgNPs-PL by transmission electron microscopy (TEM), scanning electron microscopy (SEM), energy-dispersive X-ray spectroscopy, and inductively coupled plasma-atomic emission spectrometry (ICP-AES). And we examined the cytotoxicity of AgNPs-PL on primary human periodontal ligament fibroblasts (HPDLFs) by CCK-8 assay. Then we performed colony formation assays, SEM, and LIVE/DEAD BacLight bacterial viability staining tests, to assess the antibacterial activity of AgNPs-PL against *E. faecalis*. Finally, the penetrability of PL and CH into dentinal tubules was determined by CLSM, and teeth color evaluation after the medicaments placed in the root canal for 9 days was measured with VITA Easyshade.

## Materials and methods

### Fabrication of AgNPs poloxamer thermoreversible gel

AgNPs were synthesized through the polyphenol reduction method, as the previously described method^29^. The poloxamer gel was prepared as described in a previous study^22,30^. Briefly, P188 and P407 (BASF SE, Germany) were dissolved at different mass ratios in 166 μg/mL cold AgNPs while continuously stirring. The mixture was kept at 4 °C for 24 h to form a transparent solution of 166 μg/mL AgNPs-PL. The cell viability test described below showed that AgNPs-PL at a concentration of 0–32 μg/mL was non-toxic to primary human periodontal ligament fibroblasts (HPDLFs) through 24, 48, and 72 h exposures. We, therefore, elected to use an intermediate concentration (16 μg/mL) and the highest concentration (32 μg/mL) to synthesize AgNPs-PL. In summary, P188 and P407 were dissolved in cold distilled deionized water to make a blank poloxamer gel, which was used to dilute the AgNPs-PL to the desired concentration. The polymeric dispersion was filtered using a polycarbonate membrane (pore size 0.22 μm) and stored at 4 °C until use.

### Characterization of AgNPs poloxamer thermoreversible gel

#### Gelatin temperature

**Figure 1:**
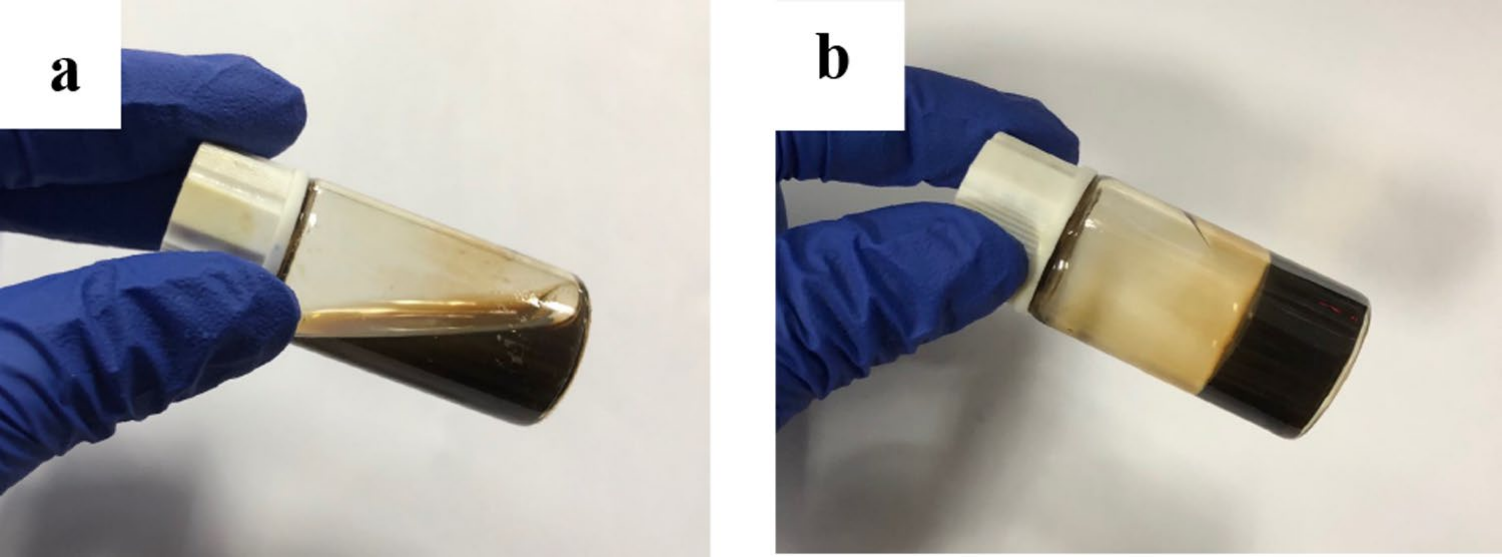
A representative photograph of the AgNPs-PL at (**a**) room temperature (23 °C) and (**b**) gelation temperature (30 °C).

The gelation temperature (Tg) of the AgNPs-PL was assessed physically by tilting at a 45-degree angle and then examining the fluidity of the gel. Vials containing 2 mL of AgNPs-PL were first incubated on an ice-cold water bath for at least 20 min to equilibrate the sample to the bath temperature before incubation on a 4 °C water bath. Subsequently, the bath temperature was gradually raised from 20 to 40 °C, and after that, by 1 °C every 5 min. At each step, the sample vials were titled over a 45-degree angle to observe gel fluidity, and the temperature was recorded. Gelation temperature was defined as the temperature at which the gel could no longer move (Fig. 1). All experiments were performed in triplicate. Samples that flowed freely at 25 ± 1 °C but not at 30 ± 1 °C, and those that exhibited short sol–gel transition times, were considered suitable for thermoreversible gels for this study. Our analysis revealed that the proper percentage ratio of P188 and P407 was 25% and 5%, respectively. Next, we prepared three gels: a control gel (P188 or P407 hydrated with distilled deionized water) and two gel formulations containing AgNPs at concentrations of 16 μg/mL and 32 μg/mL.

#### Characterization of AgNPs-PL

TEM was used to examine the morphology and size of the AgNPs-PL nanoparticles, and measure the maximum diameter of each particle. To this end, the AgNPs-PL gels were spread onto coverslips before examination using a scanning electron microscope (Tescan Vega TS5136LS). Because of the gels’ viscosity, it was difficult to observe its internal components. The cross-sections of AgNPs-PL were observed after cracking the coverslips. Energy-dispersive X-ray spectroscopy (EDAX AMETEK) (EDS) was performed at an accelerating voltage of 15 kV to determine the elemental content.

#### Ion release of AgNPs-PL

To assess the release of Ag ions from the AgNPs-PL, 20 mg of 166 μg/mL AgNPs-PL was soaked in 10 mL Tris–HCl (1 M, pH 7.4), simulated body fluid (SBF), α-MEM and brain heart infusion (BHI) for 9 days at 37 °C. The SBF solution was prepared according to the procedure described by A Yazdanpanah^31^. At the time points (1, 3, 6, and 9 days), 5 mL of the solution was collected, and the concentration of Ag^+^ released from the AgNPs-PL into the solution was quantified at each time point by ICP-AES. The withdrawn solution was replaced with 5 mL of fresh solution (Tris–HCl, SBF, α-MEM, and BHI). This analysis was done in triplicate.

#### Cell Viability of AgNPs-PL and AgNO_3_

HPDLFs (ScienCell 2630, ScienCell Research Laboratories, Carlsbad, CA) were obtained at passage 1 and supplemented with 2% fetal bovine serum (FBS) (Gibco, NY, USA), 100 U/mL penicillin, and 1% fibroblast growth supplement at 37 °C in a humidified atmosphere with 5% CO_2_ following standard aseptic techniques. When the cell growth rate reaches 70% to 80%, cell passage culture was carried out. The cells in the logarithmic growth phase were digested, collected, adjusted into cell suspension at a concentration of 1 × 10^5^/mL, and then seeded in a 96-well plate (5000 cells/well). Cell Counting Kit-8 (CCK-8) (Dojindo, Kumamoto, Japan) assays were performed to evaluate the inhibitory effects of AgNPs-PL and AgNO_3_ on HPDLFs. After incubation for 24 h, the wells were exposed to different concentrations (4, 8, 16, 32, 64 μg/mL) of the AgNPs-PL and AgNO_3_ and incubated for 24, 48, and 72 h. Meanwhile, wells containing the cell medium only were prepared for the untreated controls. After inoculation overnight, good cell adherence was confirmed via microscopic observation. Then, 10 μL CCK-8 dye was added into the culture medium and incubated for another 1.5 h at 37 °C. The optical density (OD) value was determined at 450 nm using a microplate reader (Bio-Rad, Hercules, CA). The experiment was repeated 5 times. The relative cell viability was determined by comparing the absorbance at 450 nm with the control wells that contained the cell culture medium only.

### Antibacterial effect of AgNPs-PL

#### *E. faecalis* biofilm model

We collected 191 freshly extracted human teeth with single canal and mature apical foramen due to extractions for orthodontic or periodontal reasons in the maxillofacial department of Nanjing Stomatological Hospital. This study was approved by the Ethics Committee of the School of Dental Medicine, University of Nanjing, China (ethics approval registration number: KY-2020NL-021). The methods were carried out in accordance with the Declaration of Helsinki (2008), and all patients have obtained informed consent. Firstly, we cut the teeth below the cementoenamel junction to a standardized length of 12 mm. A longitudinal groove along the center of the outer surface was created using a diamond bur. A 15# K-file was inserted into the canal until its tip was visible at the apical foramen while ensuring that the optimal working length was 0.5 mm shorter than this length. The teeth were prepared using the ProTaper NEXT system (ProTaper NEXT, Dentsply, Switzerland) up to 30# with 17% EDTA gel (File-Rite, Dentsply, Switzerland). Next, to remove the smear layer, the ultrasound instrument (P5 Newtron XS) was activated with 5.25% NaOCl for 1 min^32^. The teeth were washed with normal saline and exposed to 5.25% NaOCl and vortexed for 4 min and ended in double-distilled water for one more minute^33^. The teeth were dried with paper points, and the apical foramen was sealed with restorative glass ionomer (Fuji, GC2, Japan). Each tooth was transferred into microcentrifuge tubes containing BHI and autoclaved at 121 °C for 30 min. To verify that the teeth were sufficiently sterilized, 5 teeth were randomly chosen and cultured in BHI.

Frozen *E. faecalis* (ATCC No. 9854) were streaked onto BHI agar plates and incubated at 37 °C for 48 h. *E. faecalis* colonies were isolated, suspended in 10 mL BHI, and then incubated for 24 h. 100 μL of *E. faecalis* suspension comprising about 1.5 × 10^8^ CFU/mL was carefully injected into each canal, ensuring the bacterial suspension did not overflow. The bacteria were distributed across the whole canal using #15 K-file and then incubated for 2 weeks to allow biofilm formation in the root canal^34^. In this period, the medium was replaced with a fresh one every 2 days.

#### Root canal disinfection with different medications

Next, the teeth were randomly assigned into 4 experimental groups (n = 45). Group A: treated with PL alone group (25 g P188 and 5 g P407 dissolved in 100 mL cold de-ionized water). Group B: treated with CH paste (30% Ca(OH)_2_ paste, Metapaste, Meta Biomed, Cheongju, Korea). Group C: treated with 16 μg/mL AgNPs-PL (166 μg/mL AgNPs-PL diluted to 16 μg/mL using blank gel). Group D: treated with 32 μg/mL AgNPs -PL (166 μg/ml AgNPs-PL diluted to 32 μg/mL with blank gel).

The respective disinfectants were introduced into the canal with a 27-gauge needle, and the excess medication was removed before sealing the canal entrance with a temporary restorative material (Cavit, 3 M ESPE, Germany). The teeth were placed in a centrifuge tube with 5 mL of sterile BHI. Each treatment group was randomly split into 3 subgroups (15 samples each) and incubated for 1, 3 or 9 days. The medium was changed every 2 days. After the incubation period, the temporary fillings were removed, and each root canal was washed with 5 mL sterile saline.

#### Bacterial colonization examination

Following the treatment of the canals as described in the above section, bacteria sampling was done with sterile paper points (ProTaper NEXT paper points, Dentsply, Switzerland). 30# sterile paper points were soaked in sterile saline and placed in each root canal for 1 min, after which they were placed in microcentrifuge tubes containing 1 mL BHI and then vortexed for 1 min. The bacterial suspension was then diluted tenfold in 10 sequential steps, and 100 μL of each dilution was streaked on BHI agar plates and incubated for 24 h at 37 °C. After culturing for 48 h, the number of viable bacteria in the canal were calculated.

Next, the teeth were split longitudinally and one half randomly selected for morphological and bacteria distribution assessment using SEM. The other half was stained with LIVE/DEAD BacLight Bacterial Viability Kit (Molecular Probes, Life Technologies, Australia) containing SYTO 9 (Molecular Probes, Eugene, OR) and propidium iodide for 15 min and then rinsed with PBS according to the manufacturer’s instructions. Besides, according to the laser confocal characteristics, we also set up two additional groups, each group has three teeth: positive control (no medicaments performed in *E. faecalis*-contaminated root canals) and negative control (neither *E. faecalis* contamination nor medicaments were performed). The specimens were mounted on glass slides and a stack of 20 slices with a 0.5-mm step size was acquired for each confocal laser scanning microscopic scan were examined with CLSM (Nikon A1 Si; Nikon Corporation, Tokyo, Japan). Excitation and emission wavelengths were 480/500 nm for SYTO 9 and 490/ 635 nm for PI, respectively. For CLSM analyses: the killing rates were determined using the formula: red intensity/ (red intensity + green intensity) × 100.

### Dentinal tubule penetration of CH, PL

In the study, 40 human mandibular premolars were selected that had been extracted for the reason of orthodontic. Teeth were cut with a diamond disc under water cooling to obtain a standardized length of 16 mm. The working length was established 0.5 mm shorter from the apical foramen. The teeth were prepared using the ProTaper NEXT system (ProTaper NEXT, Dentsply, Switzerland) up to 30# with 17% EDTA gel (File-Rite, Dentsply, Switzerland). Next, to remove the smear layer, the ultrasound instrument (P5 Newtron XS) was activated with 5.25% NaOCl for 1 minute. Then the canals were dried with paper points (ProTaper NEXT paper points, Dentsply, Switzerland). The specimens were randomly distributed into 4 groups (n=10) according to the intra-canal medicament and the irrigation procedures used: group CH, group PL, group CH+PUI, and group PL+PUI. For CH and PL group, the canals were filled with medicaments mixed with 0.1% rhodamine B (Sigma-Aldrich, St Louis, MO). The prepared medicaments were delivered through the root canals using a size #30 Lentulo spiral (Dentsply Maillefer). Then the access cavities were sealed with a small cotton pellet and temporary filling material (Cavit, 3M ESPE, Germany). The specimens were incubated at 37 °C in 100% relative humidity for 2 weeks. For CH+PUI and PL+PUI group, after the incubation period of 2 weeks, the medicaments were removed with ProTaper F4 and #40 H files (Dentsply Maillefer), and the canals were irrigated with 5.25% NaOCl, which was activated with the passive ultrasonic device (P5 Newtron XS) for 1 minute.

The specimens were embedded into resin blocks and sectioned transversely using a 0.4 mm thick diamond disk at low-speed under water cooling (Isomet 1000, Buehler, IL, USA). Three sections of 1 mm thick were obtained at a distance of 3, 5, and 8 mm from the apex. Then the sections were polished with silicon carbide abrasive paper to produce a smooth surface and eliminate dentinal debris generated during the cutting procedures. All the specimens were mounted onto glass slides and examined with CLSM. The wavelengths of absorption and emission for rhodamine B of 540/590 nm and fluorescein of 536/617 nm.

The images were imported into Image J analysis software (National Institutes of Health). Dentinal tubule penetration percentage was measured using the formula: the circumference of the medicament penetration areas/the circumference of the root canal wall.

### Color evaluation

20 teeth with complete crowns were extracted and stored in sterile saline. The debris and surface pigments were removed with an ultrasonic scaler. The samples were randomly divided into 4 groups (n = 5): the PL group, the CH group, the 16 μg/mL AgNPs-PL group, and the 32 μg/mL AgNPs-PL group. The color values of the samples were measured by a digital tooth shade determination device (VITA Easyshade Compact, VITA Zahnfabrik, Bad Sackingen, Germany) in the same room. After the initial color measurement, the teeth were prepared with ProTaper NEXT system and filled with the above medicaments. And the change in color was measured at each time node (1, 3, and 9 days). The color assessment was reported using the L*a*b* system. In each analysis, the evaluation was performed in three replicates for each tooth and then averaged. Next, the tooth discoloration (∆E) was calculated according to the equation of ΔE* = ([L*1-L*0]^2^ + [a*1-a*0] ^2^ + [b*1-b*0] ^2^)^1⁄2^, where L* denotes the lightness ranging from black to white, a* indicates the redness/greenness, and b* shows blueness/yellowness. Values that are clinically acceptable for color changing and perceived by the human eye are around 3.3 for ∆E*. The color change values between 1 day and initial color baseline, 3 day and initial color baseline, 9 day and initial color baseline were calculated.

### Statistical analysis

Data were expressed as mean ± SD. The Non-parametric Kruskal–Wallis test was used for overall comparison, and the Mann–Whitney U test was used for pairwise comparison. The percentage of live cells (green) was estimated using the Kruskal–Wallis test, and Dunn’s test was applied for multiple comparisons. Differences among the coronal, middle, and apical thirds of similar samples were compared using the nonparametric Kruskal–Wallis test followed by the post hoc Siegel Castellan tests. The Mann–Whitney U test was used for pair-wise comparisons. In the color evaluation experiment, ΔE* were compared at different time points using a 2-way analysis of variance (ANOVA). To compare the mean values of appearance changes caused by different medicaments, a three-way ANOVA was performed on the ΔE*. All data analyses were performed using SPSS software (version 22.0; SPSS, Inc., Chicago, IL). Differences were considered as statistically significant when α = 0.05 and *P* < 0.05.

## Results

### Preparation and optimization of AgNPs poloxamer thermoreversible gel

Thermoreversible gels were formulated using the polymers P407 and P188. The thermoreversible nature of these polymers was manipulated to obtain the most optimal concentration of AgNPs for delivery into root canals at temperatures above 32 °C. Our analyses revealed that for P407 and P188, concentrations of 25% w/v and 5% w/v, respectively, possessed a gelation temperature of 29.7 ± 0.4 °C, which was considered optimal for gel preparation (Table 1, Fig. 1).

**Table 1:** Gelation temperature of gel at different concentration of P407 and P188.

Poloxamer (%, w/w)		Gelation temperature (°C)
P407	P188	
20	0	23.0 ± 0.3
20	2.5	36.5 ± 0.4
20	5	39.9 ± 0.4
20	7.5	43.9 ± 0.5
20	10	40.0 ± 0.4
25	0	21.9 ± 0.1
25	2.5	25.5 ± 0.3
25	5	29.7 ± 0.4
25	7.5	34.0 ± 0.4
25	10	36.1 ± 0.3

### Evaluation of AgNPs poloxamer thermoreversible gel

**Figure 2:**
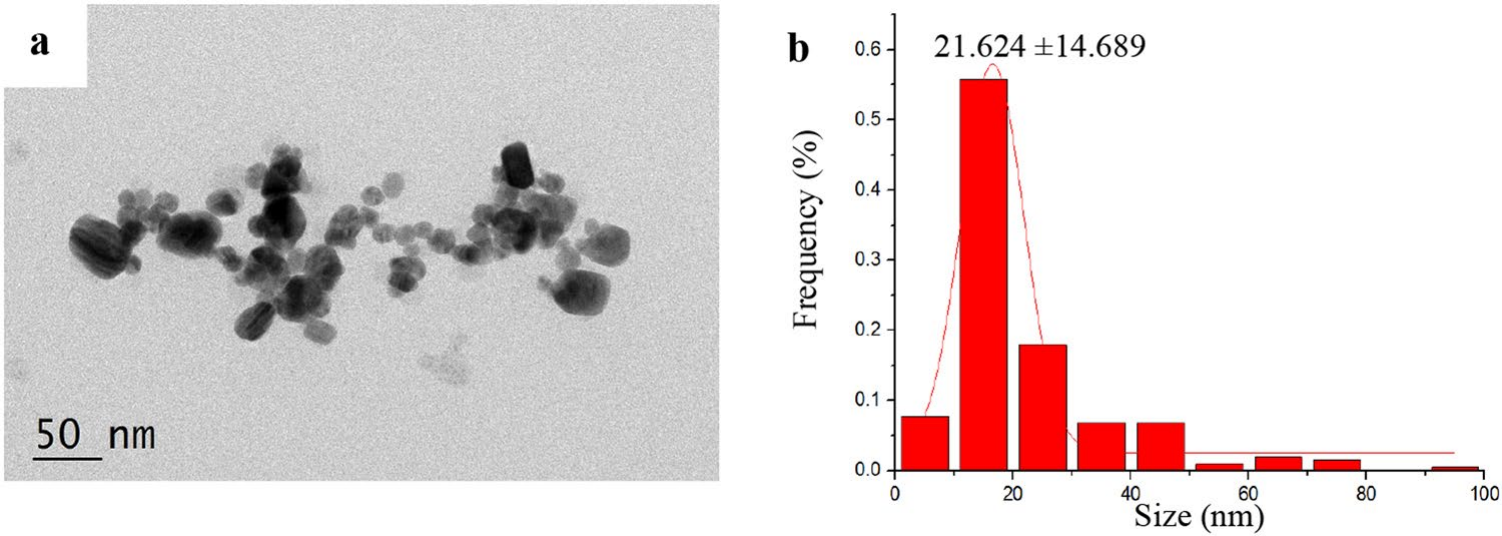
Characterization of silver nanoparticles sizes by TEM. TEM images of AgNPs-PL (**a**) and frequency of size distribution for AgNPs-PL (**b**).

**Figure 3:**
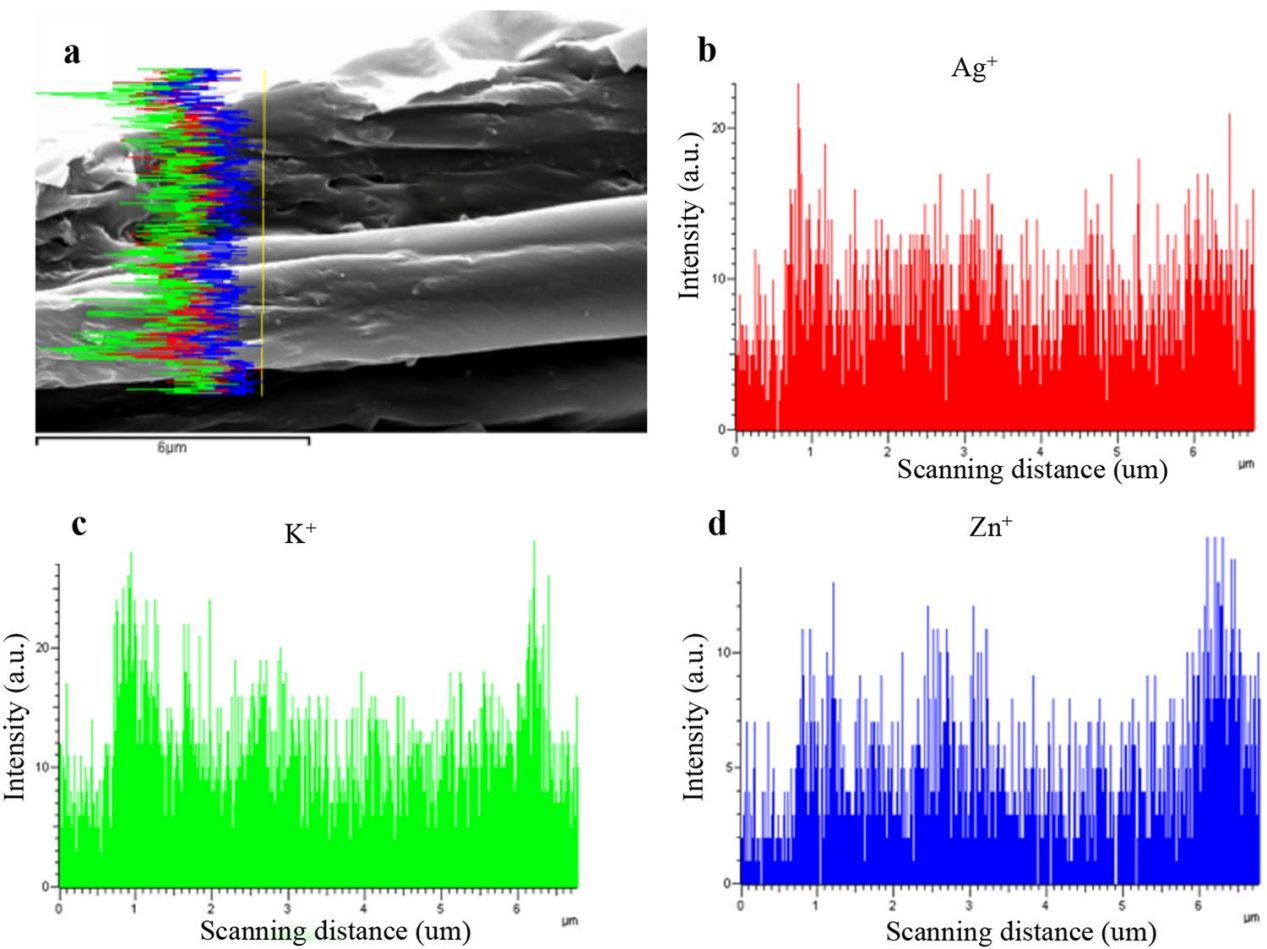
SEM micrograph (**a**) and EDS curve showing silver (**b**), potassium (**c**), zinc (**d**) distribution on coverslips of AgNPs-PL.

In our previous studies, TEM analysis revealed the sound dispersion property of nanoparticles in AgNPs solutions and that AgNPs particles are spherical and do not form any agglomerations. Besides, we found that the particle size ranged between 8–20 nm, with a mean diameter of 13.81 ± 2.21 nm^29^. In this study, TEM micrographs indicated that AgNPs-PL particles occur as large aggregates with different shapes (Fig. 2a,b), with a mean diameter of 21.624 ± 14.689 nm. Characterization of the newly prepared AgNPs poloxamer thermoreversible gels using EDS showed the presence of Ag^+^, and hence AgNPs, in gel (Fig. 3b).

### Ag^+^ release profile

**Figure 4:**
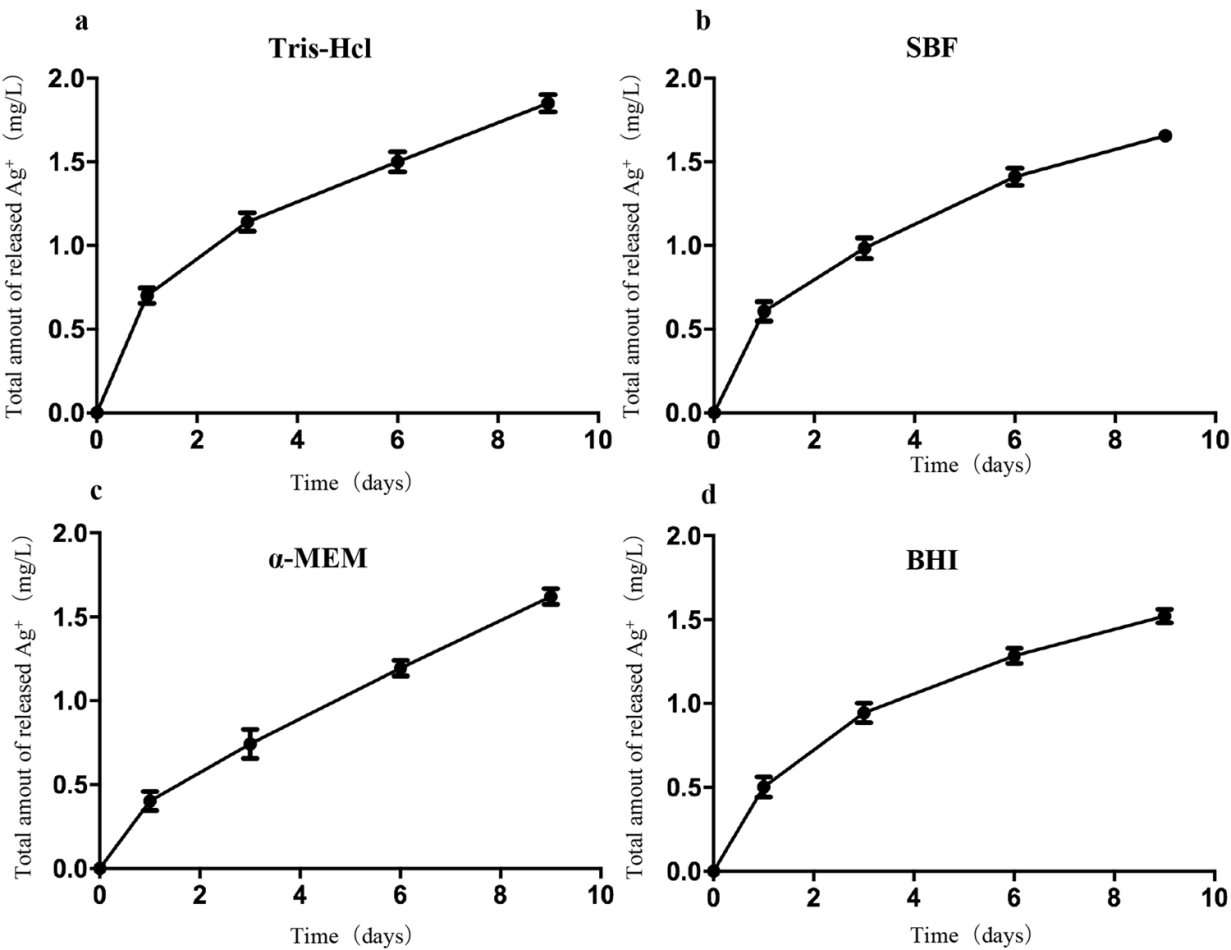
Cumulative amount of Ag^+^ ions released from AgNPs-PL in different solutions. (**a**) Ag^+^ ions released in Tris–HCL; (**b**) Ag^+^ ions released in SBF; (**c**) Ag^+^ ions released in α-MEM; (**d**) Ag^+^ ions released in BHI.

Due to the biodistribution and pharmacokinetic profile of AgNPs, continuous local release of AgNPs is not feasible^35^. To overcome this challenge, we loaded AgNPs into the poloxamer. We found that this maneuver permitted an initial release of Ag^+^ ions into different solutions (Tris–HCl, SBF, α-MEM, and BHI), which persisted for 9 days. This made this gel a capable carrier for antibacterial application to the root canal (Fig. 4), to meet the demand for antibacterial root canal medication. The amount of Ag^+^ released was highest in Tris–HCl and most stable in α-MEM (Fig. 4). A quick and constant drug release profile of Ag^+^ from the AgNPs thermosensitive hydrogel may provide potent antimicrobial activity.

### Cell viability of AgNPs-PL and AgNO_3_

**Figure 5:**
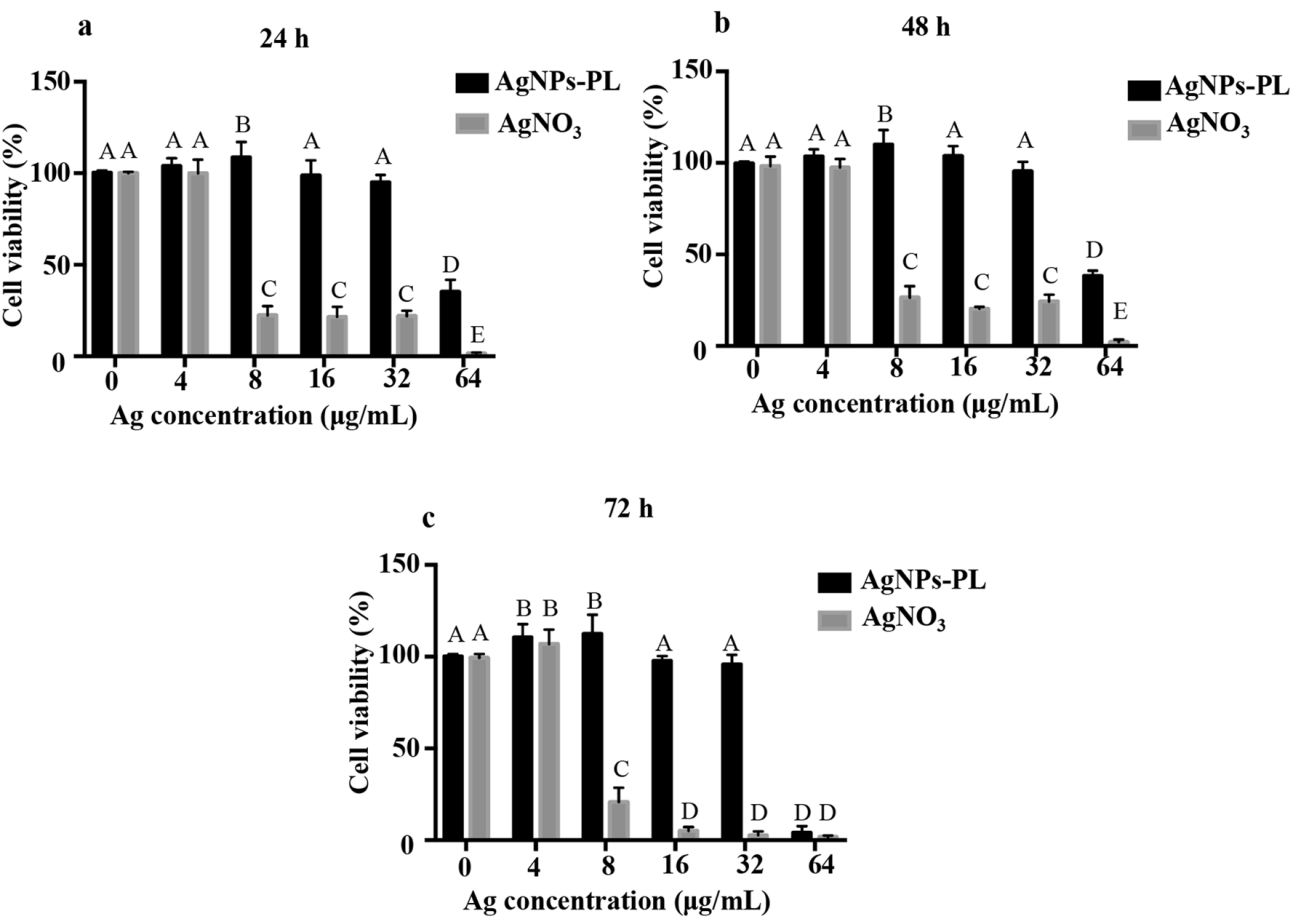
Relative cell viability of human periodontal ligament fibroblasts exposed to different densities (0–64 μg/mL) of AgNPs-PL and AgNO_3_ for 24 (**a**), 48 (**b**), and 72 h (**c**).

As shown in Fig. 5, the cell viability of HPDLFs was not affected after AgNPs-PL of low dose (4–32 μg/mL) treatment compared with untreated groups through 24, 48, and 72 h exposures (*P* > 0.05). However, the relative cell viability of 64 μg/mL AgNPs-PL significantly decreased through 24, 48, and 72 h exposures (*P* < 0.05). The difference between the AgNPs-PL group and AgNO_3_ of 8, 16, 32, 64 μg/mL was statistically significant (*P* < 0.05).

### Antimicrobial effects against planktonic *E. faecalis*

**Figure 6:**
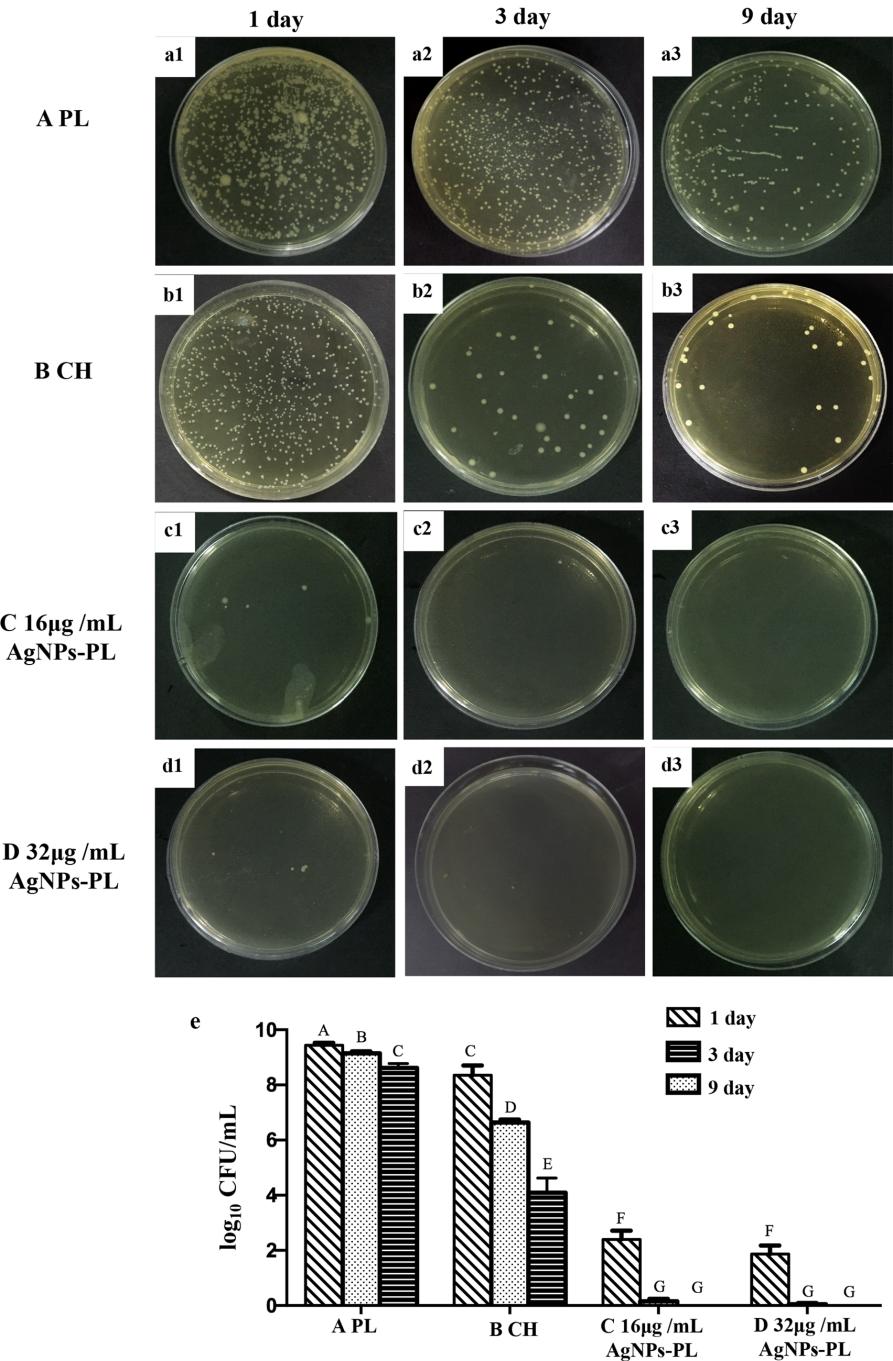
Antibacterial effects of different drugs against *E. faecalis*. (**a1**–**a3**) representative image of CFUs treated with PL for 1, 3, 9 days. (**b1**–**b3**) representative image of CFUs treated with CH for 1, 3, 9 days; (**c1**–**c3**) representative image of CFUs treated with 16 μg/mL AgNPs-PL for 1, 3, 9 days; (**d1**–**d3**) a representative image of CFUs treated with 16 μg/mL AgNPs-PL for 1, 3, 9 days; (**e**) CFU/mL counts of *E. faecalis* bacteria after treatment with different drugs for 1, 3, 9 days. Significant differences between columns are labelled by different upper-case letters (*p* < 0.05).

Analysis of antibacterial activity against planktonic *E. faecalis* over 9 days revealed that 16 μg/mL and 32 μg/mL AgNPs-PL strongly inhibited the growth of *E. faecalis* and the effects at this dosage were significantly better than those attained by the CH and negative control treatment (*P* < 0.05) (Fig. 6e). Of note, no significant differences in antibacterial activity was observed between AgNPs-PL at 16 μg/mL or 32 μg/mL AgNPs-PL (*P* > 0.05) (Fig. 6e). The CH treatment had a limited inhibitory effect on planktonic *E. faecalis* (Fig. 6b). In all groups, the amount of *E. faecalis* within the main root canal declined over time (Fig. 6a–d), possibly because the closure of the apical foramen and canal orifices blocked nutrient entry into the root canal.

### SEM observations

**Figure 7:**
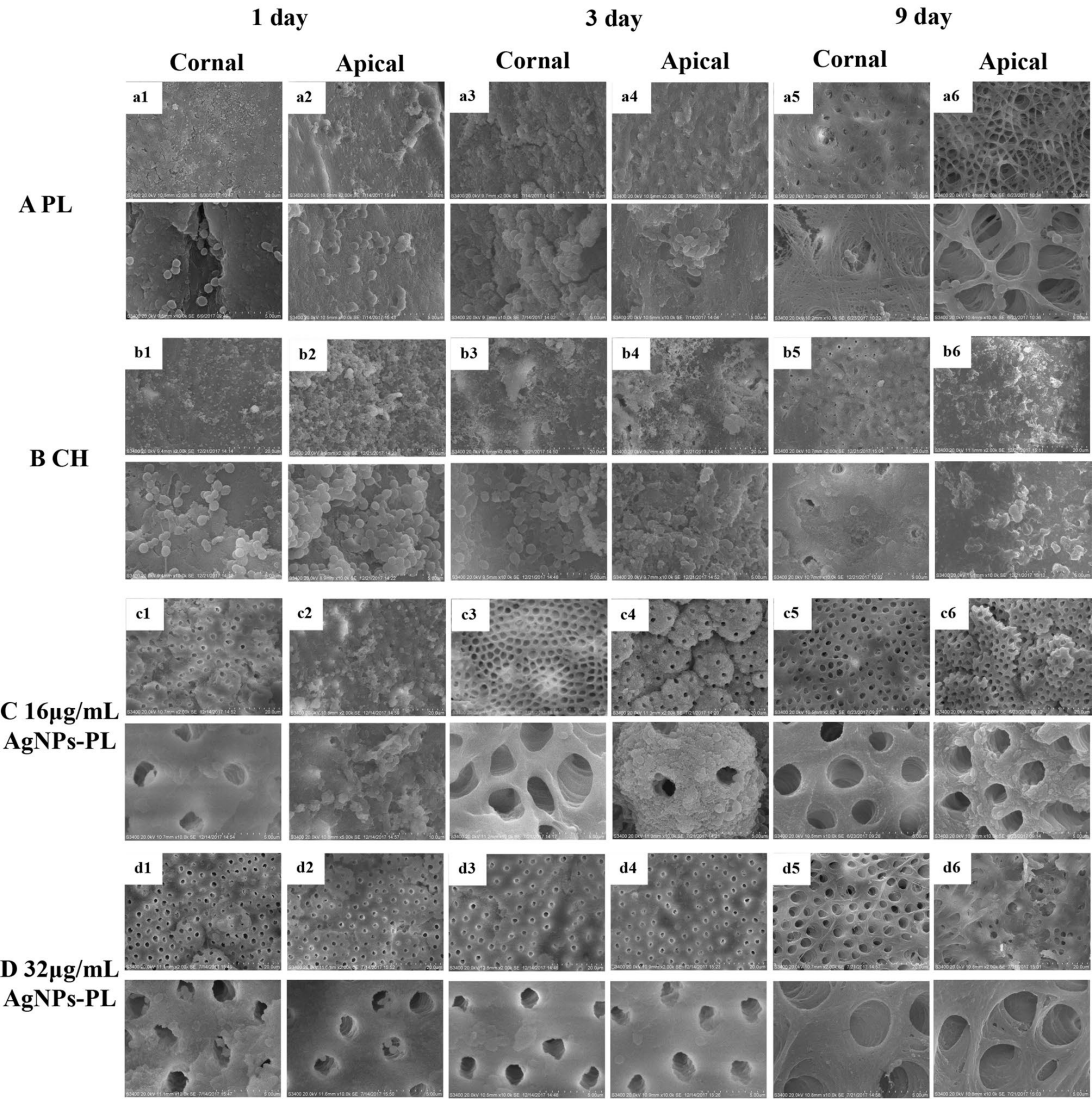
Representative field emission scanning electron microscopic images showing colonization of *E. faecalis* on root canal walls (the first row of each group × 2000 magnification; the second row of each group × 10,000 magnification). (**a1**–**a6**) representative image showing growth of *E. faecalis* on coronal and apical part of root canal wall in PL group for 1, 3, 9 days; (**b1**–**b6**) representative image showing growth of *E. faecalis* on coronal and apical root canal wall in CH group for 1, 3, 9 days; (**c1**–**c6**) representative image showing growth of *E. faecalis* on coronal and apical root canal wall in 16 μg/mL AgNPs-PL group for 1, 3, 9 days; (**d1**–**d6**) representative image showing growth of *E. faecalis* on coronal and apical root canal wall in 32 μg/mL AgNPs-PL group for 1, 3, 9 days.

SEM images revealed differences in bacteria growth on the coronal and apical root canal walls among the groups. SEM analysis of split-half roots revealed numerous *E. faecalis* cells colonizing the root canal wall and growing into dentinal tubules in the PL group at day 1, day 3, and day 9 (Fig. 7Aa1–a6). A similar observation was made for the CH group at day 1 and day 3 (Fig. 7Bb1–b4). *E. faecalis* cells form biofilms and aggregate into grape-like colonies, with a large number of mycelial junctions between cells (Fig. 7Bb1–b4). At day 9, *E. faecalis* biofilms were still present on the root canal walls treated with CH, but they lacked mycelial connections between cells (Fig. 7Bb5,b6). In the CH group, calcium hydroxide particles covered the surface of the dentin wall, constricting the dentinal tubule, which made it difficult to observe (Fig. 7Bb1–b6). Only a small proportion of *E. faecalis* was observed in the coronal and apical parts of canals following treatment with AgNps-PL at 16 and 32 μg/mL for 3 days (Fig. 7Cc3–c4), (Fig. 7Dd31–d4) and no *E. faecalis* bacteria were observed after 9 days (Fig. 7Cc5–c6), (Fig. 7Dd5–d6). In addition, dentinal tubules were visible throughout the 9-day treatment with AgNps-PL at 16 and 32 μg/mL group (Fig. 7Cc1–c6,Dd1–d6).

### CLSM analysis

**Figure 8:**
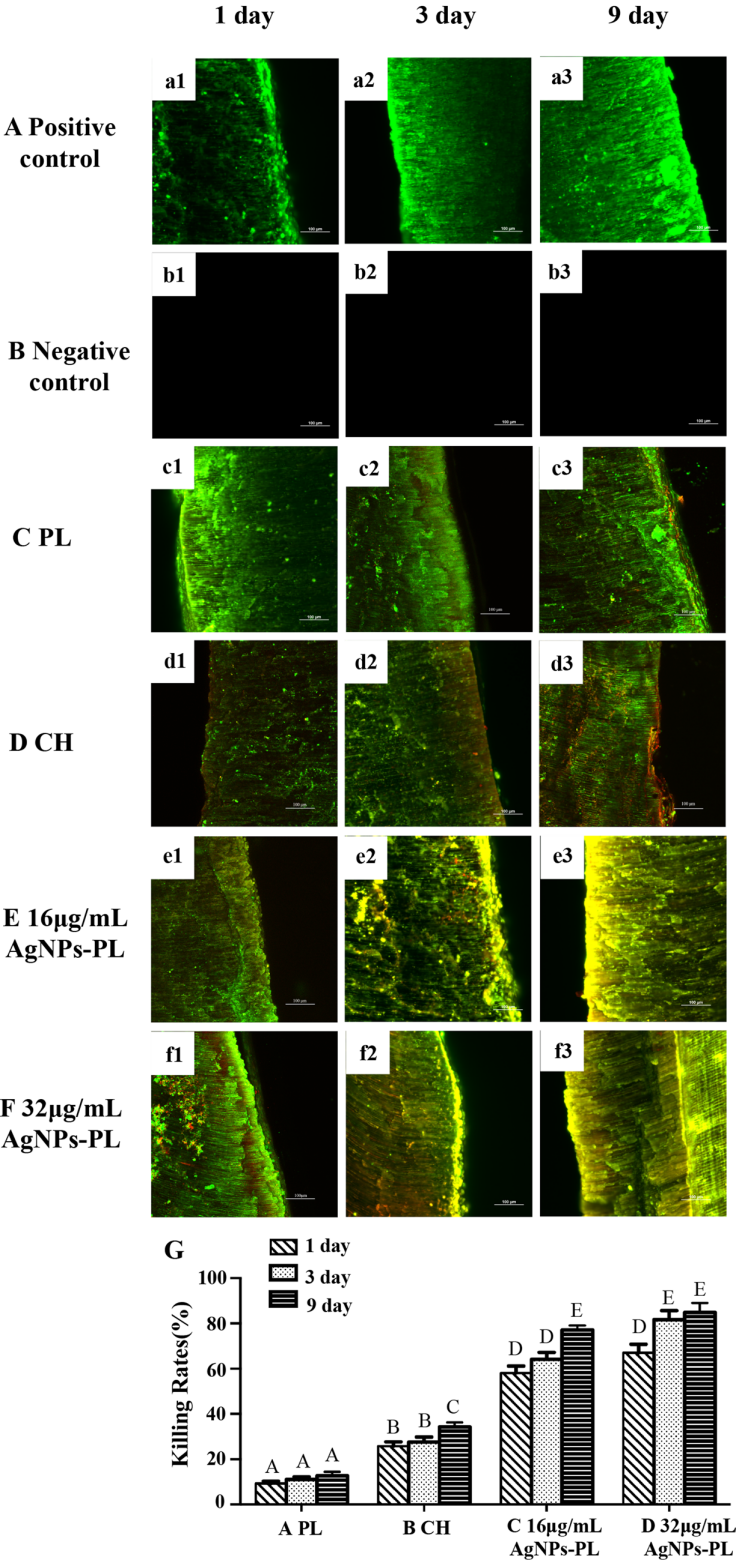
Representative live/dead bacterial staining images of the split dentin infected with *E. faecalis* and then treated with different drugs: (**a1**–**a3**) PL group; (**b1**–**b3**) CH group; (**c1**–**c3**) 16 μg/mL AgNPs-PL group; (**d1**–**d3**) 32 μg/mL AgNPs-PL (scale bar: 100 μm); (**e**) Comparison of killing rates of different agents. Live bacteria were stained green, and dead bacteria were stained red. Significant differences between columns are labeled by different upper-case letters (*p* < 0.05).

Next, to detect live/dead bacteria, we carried out CLSM analysis on the dentinal tubules. Figure 8 shows representative images of live/dead bacteria in dentinal tubules after treatment with the 4 agents. In descending order, the reduction of *E. faecalis* at 1 day after treatment was as follows: 16 μg/mL AgNPs-PL (58.08%), 32 μg/mL AgNPs-PL (66.99%) > CH (25.75%) > PL (9.311%) (*P* < 0.05). The reduction of *E. faecalis* in the 3-day after treatment was as follows: 32 μg/mL AgNPs-PL (71.12%) > 16 μg/mL AgNPs-PL (64.16%) > CH (27.58%) > PL (11.09%). After treatment for 9 days, the reduction in *E. faecalis* was as follows: 32 μg/mL AgNPs-PL (84.88%), 16 μg/mL AgNPs-PL (77.08%) > CH (34.35%) > PL (12.65%) (Fig. 8G).

### Dentinal tubule penetration of CH, PL

**Figure 9:**
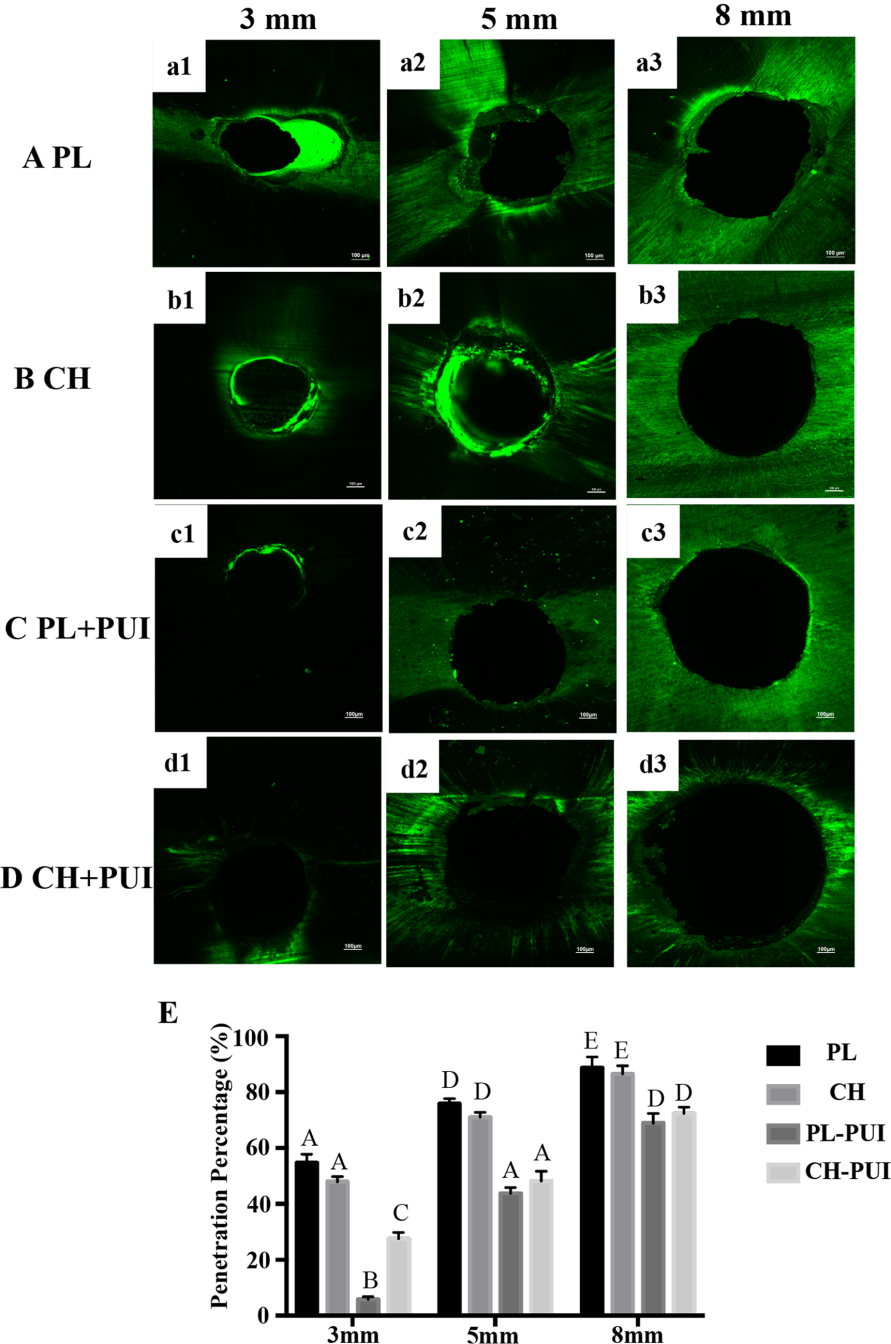
Representative confocal laser scanning microscopic images from each group at coronal, middle and apical thirds: (**a1**–**a3**) PL group; (**b1**–**b3**) CH group; (**c1**–**c3**) PL-PUI group; (**d1**–**d3**) CH-PUI group (scale bar: 100 μm); (**d**) Penetration percentage of the PL and CH into the dentinal tubules in all experimental groups. Significant differences between columns are labelled by different upper-case letters (*P* < 0.05).

No significant differences were found among PL and CH sections concerning the depth reached by the medicaments into the dentinal tubules at 3, 5, and 8 mm from the root apex (*P*> 0.05) (Fig. 9). When comparing the sections of PL and CH made at 3, 5, and 8 mm after PUI, the Penetration Percentage of the PL-PUI was significantly lower than the CH-PUI group at 3 mm (*P*<0.05) (Fig. 9).

### Color evaluation

**Figure 10:**
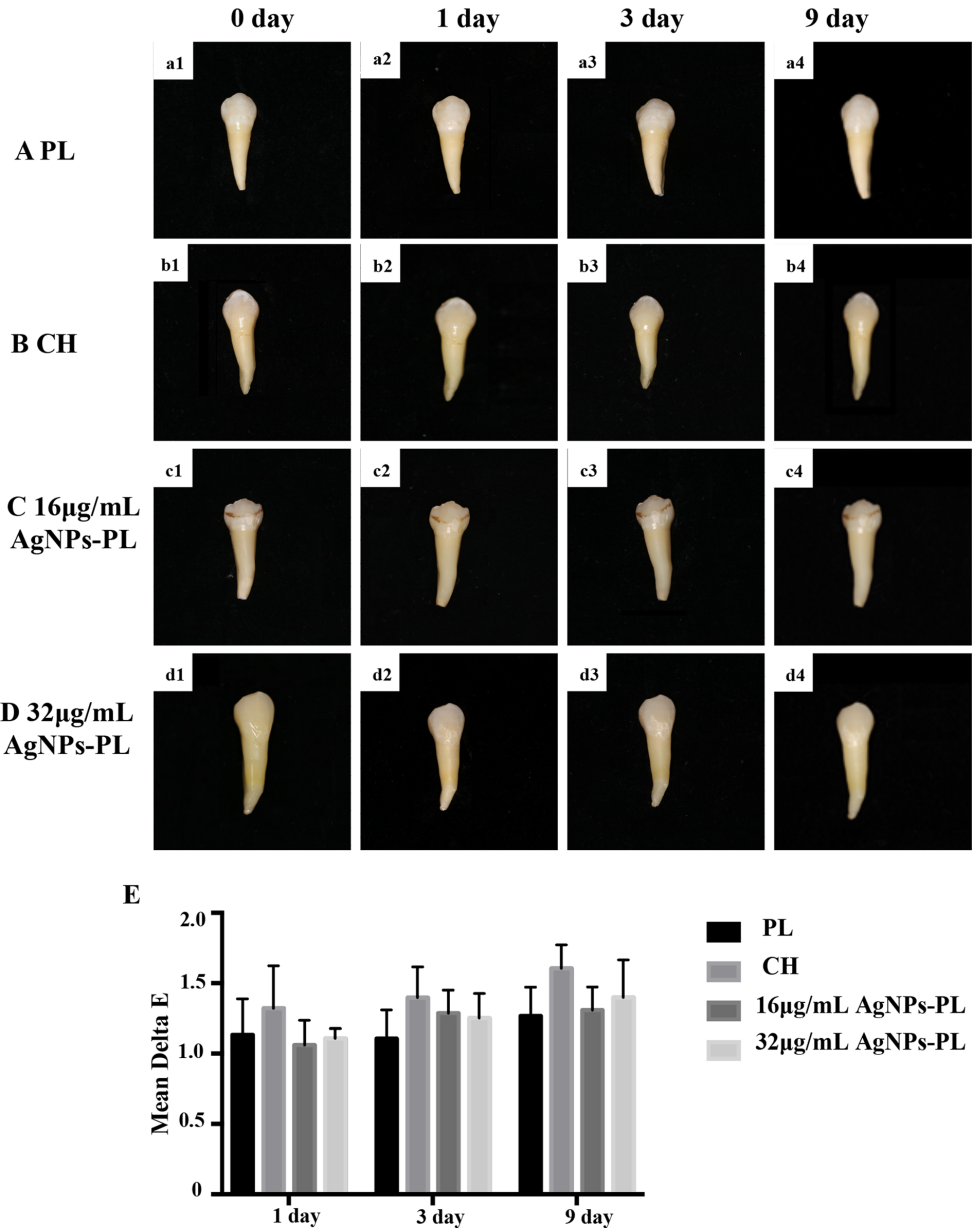
Photographic images of the 0, 1, 3, and 9 days measurements. (**a1**–**a4**) PL group; (**b1**–**b4**) CH group; (**c1**–**c4**) 16 μg/mL AgNPs-PL group; (**d1**–**d4**) 32 μg/mL AgNPs-PL; e. ∆E* value after treatment with different medicaments for 0, 1, 3, and 9 days.

Based on the findings of Fig. 10, there was no significant difference in the discoloration rate after 1, 3, 9‐day treatment between different medicaments (*P*> 0.05). In addition, the ∆E* value of all groups was less than 2.0, which means no color change visible for the human eye.

## Discussion

Poloxamer has been widely used as a nonionic stabilizer due to its low toxicity and relatively fast dissolution rate^21,36,37^. This compound can form a hydrogel, which is a 3D cross-linked polymer network that absorbs vast amounts of water^38^. In this study, we designed and characterized gels containing AgNPs. We then added P407 and P188 to the gels to make them thermally reversible and evaluated the influence of the change in the concentration of P407 and P188 on gelation temperature. The gelation temperature of 29.7 ± 0.4 °C was selected as being most suitable for use in root canals. The mass ratio of P407 and P188 was 25/5 (%, w/w) (Table 1).

In recent years, AgNPs have been demonstrated to possess excellent antibacterial properties owing to their unique nanoparticle features, including small size effects, quantum effects, and specific surface area^15,39^. In addition, there is a lower likelihood of bacteria developing resistance to AgNPs as they frequently do to antibiotics^40^. AgNPs disrupt various bacterial cell components, including the cell membrane, plasmids, and enzymes^41,42^. AgNPs offer significant specificity with improved bioavailability compared with existing conventional therapeutic agents^43^. In this study, we synthesized spherical and monodisperse AgNPs of approximately 21.6 nm (Fig. 2) and dispersed them in the poloxamer gel for examination by TEM (Fig. 3). However, due to its biodistribution and pharmacokinetic characteristics, continuous local release of AgNPs is difficult to achieve^35^. To overcome this challenge, we loaded AgNPs into the poloxamer and tested the cumulative amount of released Ag^+^ ions in Tris–HCl, SBF, α-MEM, and BHI. Our analyses revealed that Ag^+^ ions are released at a sustained rate of over 9 days. The sustained release of Ag^+^ ions from AgNPs-PL is desired as it is expected to prolong and sustain the antimicrobial action. The observed sustained release of Ag^+^ is consistent with observations we made in endodontic antibacterial tests in the root canal. To the best of our knowledge, this is the first study that compares the anti-biofilm efficacy of CH with AgNPs-PL that can release Ag^+^ sustained.

Root canal medicaments must have low cytotoxicity to be used clinically. For this reason, in vitro cytotoxicity was evaluated using the CCK-8 assay. HPDLFs represent an appropriate model for testing cytotoxicity of root canal medicaments^44,45^. Moreover, the normal diploid cells (e.g. fibroblasts), because of the relative similarity to in vivo conditions, are preferred to other established cell lines^44^. When 0–64 μg/mL AgNPs-PL was applied to HPDLFs for 24, 48, and 72 h, low dose (0–32 μg/mL) AgNPs-PL showed greater biocompatibility with HPDLFs. However, when the concentration was increased to 64 μg/mL, the AgNPs-PL showed significant cell toxicity at 72 h. Therefore, 16 μg/mL and 32 μg/mL AgNPs-PL were selected for subsequent antibacterial tests. Here, we evaluated the effectiveness of three intracanal medications (CH, 16 μg/mL AgNPs-PL, and 32 μg/mL AgNPs-PL) against *E. faecalis*.

The formation of microbial biofilms is a survival mechanism through which microorganisms develop a powerful protective structure against antimicrobial drugs, making the microorganisms difficult to treat through conventional methods^46^. In this study, we incubated *E. faecalis* for 14 days and used it as a model endodontic biofilm. This bacterial was chosen because it has a high occurrence (90%) in treated root canals with periapical periodontitis and is resistant to many antibacterial agents^47–49^. Differences inhibition effects were observed in groups treated with PL, CH, 16 μg/mL AgNPs-PL and 32 μg/mL AgNPs-PL. Conventional intracanal disinfectants like CH showed compromised antibacterial effects, due to the release and diffusion of hydroxyl ions to create a strongly alkaline environment^10,50^. However, our results suggest that CH is incapable of eliminating *E. faecalis* on the root canal surface and in dentinal tubules after exposure for 1, 3, and 9 days. This might be caused by (1) *E. faecalis* developing resistance to strong alkalinity and (2) low solubility and diffusivity of CH particles, which severely limits the ability of CH to adhere to and penetrate dentinal tubules^10,51,52^. Our laser confocal examination revealed that the red fluorescence of the CH group is more concentrated on the wall of the root canal, but lower within dentinal tubules. They are suggesting that CH does not efficiently penetrate dentinal tubules. The CH limitations identified here are similar to those reported by previous studies^46,53^.

Because of strong antibacterial properties, there has been growing interest in researching the effects of AgNPs against oral pathogenic bacteria, as well as their use for sustained drug delivery in the treatment of oral infections^54–56^. Elif Ertem and colleagues synthesized core–shell AgNPs and used them to develop two irrigation solutions that effectively removed the smear layer from the dentin surface and exhibited extended antimicrobial activity for more than 7 days^57^. Afkhami F et al. found that when AgNPs were used in combination with CH as intracanal medication, they significantly reduced the amounts of short-term intracanal microorganisms^58^. In our study, treatment with AgNPs-PL for 1, 3, and 9 days revealed a significantly higher reduction in the number of viable *E. faecalis* cells relative to CH and PL. Our analyses suggested that *E. faecalis* adhering to the root canal wall can be eradicated by a 9-day treatment with 16 μg/mL and 32 μg/mL AgNPs-PL (Fig. 7C,D). For *E. faecalis* in dentine tubules, the killing rates for following 32 μg/mL treatment with AgNPs-PL for 3 days was significantly higher than that of 16 μg/mL AgNPs-PL (Fig. 8). The antibacterial activity of 16 μg/mL and 32 μg/mL AgNPs-PL to *E. faecalis* in the main root canal and dentine tubule, was significantly higher than that of CH. Taken together, these observations indicate that AgNPs-PL with templated nanosilver might possess stronger antibacterial ability than the conventionally used CH. As an intracanal medicament, AgNPs-PL has the characteristics of low cost and easy preparation, and its superior antibacterial property has the potential of enhancing the endodontic treatment efficacy.

It was found that the penetration depth of the bacteria in the dentinal tubules can reach up to 1000 μm^59^. Intracanal medicaments should penetrate deeply and compactly through dentinal tubules to exert antibacterial and blockage effect to prevent reinfection^60^. In this study, the penetration of CH and PL into dentin tubules was analyzed by CLSM. Compared with scanning electron microscopy (SEM) technology, CLSM technology has the advantages of standard, repeatability, rapid, objective, and does not destroy the sample to generate three-dimensional images^61^. In the present study, no significant difference was found among PL and CH sections concerning the depth reached by the medicaments into the dentinal tubules at 3, 5, and 8 mm from the root apex. It may be considered from the side that the antibacterial strength of AgNPs-PL was stronger than that of CH, not because of its better permeability, but because of the antibacterial effect of Ag^+^. For the permeability of medicaments in different parts of the root canal, it was found that both CH and PL groups had the lowest penetration percentage in the apical segment. Anatomical characteristics affect medicament penetration. Compared with the middle third and cervical third, the dentinal tubules in the apical third are smaller in diameter and density^62,63^. However, this study has some limitations in that the detection index was relatively single, for the magnification of CLSM used in this research did not allow the analysis of the maximum penetration up to the cement-enamel junction^62^. Although there are limitations in measuring the dentinal tubule penetration percentage alone, it could be seen from Fig. 9 that PL and CH have similar permeability, with penetration depths greater than 500 nm. Numerous studies have found that calcium hydroxide residues in root canal treatment affect the quality of root canal filling, dentinal bond strength, and can increase apical leakage^64–66^. Although various techniques have been proposed, none has been able to remove the CH dressing completely^65,67,68^. In this study, 5.25% NaOCl combined with a passive ultrasonic device was used to remove medicaments in the root canal, but the study found that both PL and CH could not be completely removed. Further studies are needed to fully understand whether the use of AgNPs-PL in root canal therapy affects the penetrability of sealers into dentinal tubules and whether they affect dentinal bond strength.

Whether intracanal medicaments can cause tooth color changes is a question worthy of attention. As the most commonly used intracanal medicament, researchers have conducted a lot of research on whether CH can cause tooth discoloration. Kim et al. and Lenherr et al. demonstrated that CH did not cause significant changes in tooth color after 12 weeks, although CH resulted in yellowness and lightening of specimens^69,70^. In this study, 16 μg/mL AgNPs-PL group and 32 μg/mL AgNPs-PL group showed less discoloration compared to CH group, but not statistically different. In general, low-dose AgNPs-PL gel as a medicament did not cause any color change in the short term.

Although *E. faecalis* is the most prevalent bacterial species in cases of refractory apical periodontitis, affected canals frequently contain multiple types of microorganisms. Therefore, further studies are needed to characterize the inhibitory effect of AgNPs-PL on a variety of bacterial biofilms.

## Conclusion

In this study, we used this formulated and characterized AgNPs-PL for use in root canal therapy. The prepared gels exhibited advantages of sustained release of Ag^+^ and strong anti-biofilm of *E. faecalis* for 9 days. Thus, AgNPs-PL at 16 μg/mL and 32 μg/mL has excellent potential for clinical use to eliminate *E. faecalis* biofilms on dentine and in dentinal tubules. However, further studies are required to determine whether AgNPs-PL is effective against other types of microorganisms.
